# The Dancing *Taenia* Sign

**DOI:** 10.4269/ajtmh.25-0741

**Published:** 2026-04-14

**Authors:** Emiri Muranaka, Yuichi Hasegawa, Yasuyuki Morishima, Ryota Hase

**Affiliations:** ^1^ Department of Infectious Diseases, Japanese Red Cross Narita Hospital, Narita, Japan;; ^2^ Department of Laboratory Medicine, Japanese Red Cross Narita Hospital, Narita, Japan;; ^3^ Department of Parasitology, National Institute of Infectious Diseases, Japan Institute for Health Security, Shinjuku, Japan

A 34-year-old Japanese man presented with a 3-month history of passing motile proglottids. He had traveled to Indonesia, where he repeatedly consumed self-prepared raw beef (steak tartare). Abdominal ultrasonography revealed a double-lined, hyperechoic linear structure with rhythmic undulating motion in the small bowel lumen (Figure 1; Supplemental Video 1). This characteristic appearance was identified as the “dancing *Taenia* sign,” which strongly suggested a live tapeworm infection.^1^

**Figure 1.**
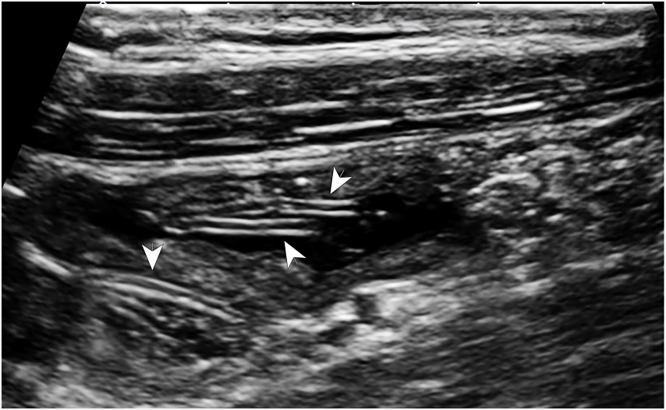
Abdominal ultrasonography revealing the characteristic double-lined hyperechoic linear structure of *Taenia* sp. within the small bowel (arrowheads).

A stool examination subsequently confirmed the presence of *Taenia* eggs, and genetic analysis of the expelled proglottids was conducted to identify *Taenia saginata* (*T. saginata*; Figure 2). The patient was successfully treated with a single 600 mg dose of praziquantel.^2^

**Figure 2.**
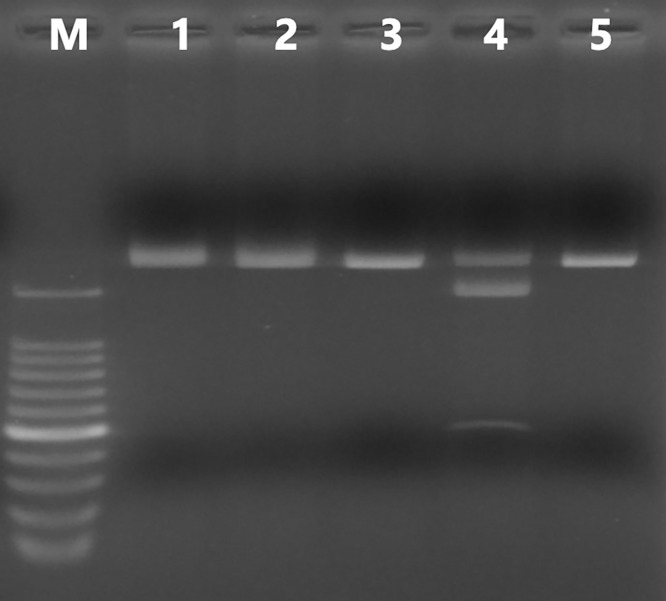
Agarose gel electrophoresis of PCR-RFLP analysis of the *cox1* gene. The polymerase chain reaction product was only cleaved by restriction enzyme *Taq*I-v2, but not by *Bam*HI, *Dde*I, or *Nco*I, indicating the species *Taenia saginata*. Lane M: 100 bp DNA ladder; Lane 1: BamHI; Lane 2: DdeI; Lane 3: NcoI; Lane 4: TaqI-v2; Lane 5: Control.

Although *T. saginata* infection is rare in Japan and predominantly imported,^3^ the present case underscores the utility of ultrasonography. The modality enabled rapid visualization of the live worm’s motion, which is distinct from the static “four-lines sign”^4^ of *Ascaris lumbricoides*, facilitating prompt diagnosis before stool results were available.^1^ Real-time ultrasonography provides a noninvasive means to visualize intestinal parasites and should be considered in travelers returning from endemic regions with relevant dietary history or clinical signs.^3^

## Supplemental Materials

10.4269/ajtmh.25-0741 Supplemental Materials

## References

[1] Fabijanić D Giunio L Boršić D Vidjak V Ledinsky M Bezić J, 2001. Ultrasound detection of intestinal taeniasis. J Ultrasound Med 20: 275–277. 11270533 10.7863/jum.2001.20.3.275

[2] Centers for Disease Control and Prevention, 2024. *Clinical Overview of Taeniasis*. Available at: https://www.cdc.gov/taeniasis/hcp/clinical-overview/index.html. Accessed December 9, 2025.

[3] Tsuboi M Hayakawa K Yamasaki H Katanami Y Yamamoto K Kutsuna S Takeshita N Kanagawa S Ohmagari N Kato Y, 2018. Clinical characteristics and epidemiology of intestinal tapeworm infections over the last decade in Tokyo, Japan: A retrospective review. PLoS Negl Trop Dis 12: e0006297. 29462133 10.1371/journal.pntd.0006297 PMC5834203

[4] Mahmood T Mansoor N Quraishy S Ilyas M Hussain S, 2001. Ultrasonographic appearance of *Ascaris lumbricoides* in the small bowel. J Ultrasound Med 20: 269–274. 11270532 10.7863/jum.2001.20.3.269

